# Morphological analysis of *Trichomycterus areolatus* Valenciennes, 1846 from southern Chilean rivers using a truss-based system (Siluriformes, Trichomycteridae)

**DOI:** 10.3897/zookeys.695.13360

**Published:** 2017-09-05

**Authors:** Nelson Colihueque, Olga Corrales, Miguel Yáñez

**Affiliations:** 1 Laboratorio de Biología Molecular y Citogenética, Departamento de Ciencias Biológicas y Biodiversidad, Universidad de Los Lagos, Avenida Alcalde Fuchslocher 1305, Casilla 933, Osorno, Chile; 2 Departamento de Estadística, Universidad del Bío-Bío, Casilla 5-C, Concepción, Chile

**Keywords:** Morphological variability, morphometry, multivariate analysis, *Trichomycterus areolatus*, truss-based system

## Abstract

*Trichomycterus areolatus* Valenciennes, 1846 is a small endemic catfish inhabiting the Andean river basins of Chile. In this study, the morphological variability of three *T. areolatus* populations, collected in two river basins from southern Chile, was assessed with multivariate analyses, including principal component analysis (PCA) and discriminant function analysis (DFA). It is hypothesized that populations must segregate morphologically from each other based on the river basin that they were sampled from, since each basin presents relatively particular hydrological characteristics. Significant morphological differences among the three populations were found with PCA (ANOSIM test, *r* = 0.552, *p* < 0.0001) and DFA (Wilks’s λ = 0.036, *p* < 0.01). PCA accounted for a total variation of 56.16% by the first two principal components. The first Principal Component (PC1) and PC2 explained 34.72 and 21.44% of the total variation, respectively. The scatter-plot of the first two discriminant functions (DF1 on DF2) also validated the existence of three different populations. In group classification using DFA, 93.3% of the specimens were correctly-classified into their original populations. Of the total of 22 transformed truss measurements, 17 exhibited highly significant (*p* < 0.01) differences among populations. The data support the existence of *T. areolatus* morphological variation across different rivers in southern Chile, likely reflecting the geographic isolation underlying population structure of the species.

## Introduction

Almost all species display morphological variation within and among populations in response to environmental and genetic factors, or as a consequence of behavioral and physiological differences (West–Eberhard 1989, Schwander and Leimar 2011). The effects of genetic factors on morphological variations have been well documented in natural populations of several fish species (e.g. Keeley et al. 2006, Taylor et al. 2011, Reid and Peichel 2010). Environmentally-induced morphological variation, or phenotypic plasticity (West–Eberhard 1989), has also been reported in fishes (Pakkasmaa and Piironen 2000, Reis et al. 2006, Bagherian and Rahmani 2009, Mir et al. 2013). In particular, hydrological condition of rivers may play an important role in the body shape changes of fishes. For example, water velocity could have a significant effect on different attributes of body shape, such as, head depth and length, caudal peduncle depth, caudal fin depth and length, and body depth, among others (Imre et al. 2002, Keeley et al. 2006, Grünbaum et al. 2007, Istead et al. 2015). These findings indicate that fishes are largely amenable to environmentally-induced morphological variations.

*Trichomycterus* genus is an interesting group of catfishes for the studying morphological variation because most species have a wide distribution in different habitats across a broad altitudinal and latitudinal range in South America. *Trichomycterus* belongs to the family Trichomycteridae, which is native to southern Central and South America (Berra 1981, Arratia 1990) and includes eight sub-families, 40 genera and >170 valid species (Eschmeyer et al. 2016). The particular biogeography of these catfishes often results in numerous isolated, slightly-differentiated populations, denoted by a marked intraspecific variation (Arratia 1990). However, the factors driving morphological variation in this group remain poorly understood.

In Chile, *Trichomycterus* is represented by five endemic species (Pardo et al. 2005), with *Trichomycterus areolatus* Valenciennes, 1846 (Siluriformes, Trichomycteridae) being a species characterized by inhabiting the rhithronic zone of freshwater systems of the Andean river basins (Arratia 1981, Vila et al. 1999). This small catfish, of less than 10 cm in length, inhabits a wide latitudinal and altitudinal range across the country, from 28 to 42°S (or a distance of about 1,500 km), and between zero and 4,000 meters above sea level, respectively (Vila et al. 1999, Dyer 2000, Unmack et al. 2009a). In addition, reproduction, feeding and shelter activities of this catfish regularly occur in habitats characterized by shallow water with a substratum of small stones with fine sand where there is rapid water velocity, located in the rhithronic zones of rivers (Arratia 1983, Campos 1985, García et al. 2012). In comparison with other sympatric freshwater fishes, either native or non-native, inhabiting Chilean river basins, ecological studies indicate that *T. areolatus* is relatively more abundant (Ruiz et al.1993, Ruiz 1996, Valdovinos et al. 2012).

Morphological studies of *T. areolatus*, which have focused mainly on northern and central Chilean populations, indicated intraspecific variation for some morphological characters. Variation has been documented in the bones of caudal skeleton (Arratia et al. 1978, Arratia 1982, 1983) and in geometric characters at the head region (Pardo 2002). The origin of morphological variation in *T. areolatus* remains unknown, but potential causes include local adaptation, due to the environmental variability of rivers, or geographic isolation of populations due to the physical separation between hydrographic basins (Pardo 2002). Another possible source of variation is the marked genetic structure of this species throughout its distribution range, associated with the low gene flow among drainage systems (Unmack et al. 2009a; Quezada–Romegialli et al. 2010). As studies undergone to date have mainly targeted northern and central regions of its range; it is unclear whether southern Chilean populations of *T. areolatus* exhibit similar levels of morphological variation. These catfish populations occupy a geographic zone of western of southern South America which, according to Hulton et al. (2002), was the site of a strong glaciation process (between latitudes 38°and 55°S) during the Last Gracial Maximum around 19,000–23,000 cal yr ago, with major ice sheets covering vast parts of the region. This geological may impacted the distribution of different species in southern Chile (Villagrán 1990). During this glaciation process, freshwater species survived in refuges and then recolonized rivers after glacial events. This process likely impacted the genetic structure of *T. areolatus* through tight bottlenecks across the distribution range, especially in basins subjected to significant ice cover during glaciation. In addition, the basins in southern Chile are made up of relatively short rivers that flow from east to west, each occupying large drainage areas. Rithonic biotopes represent about 70% of these basins (Campos 1985). In these hydrographic systems the movement of species is typically limited by connections among riverine systems, with the ocean providing an effective barrier at each river terminus. As a result of these topographic characteristics, *T. areolatus* populations between different basins are mostly isolated, which limits gene flow and promotes population subdivision. Studies to date on *T. areolatus* populations across southern Chile reveals high level of genetic divergence, which suggest limited movement between basins (Unmack et al. 2009a).

Morphometric variation between populations can provide a basis for population differentiation, which is an important tool for evaluating population structure and identifying discrete groups (Turan 1999). There are many studies on native Chilean freshwater fish that provide evidence for population or species discrimination based on traditional morphometric characters (Campos 1982, Gajardo 1987, Campos et al. 1996, Campos and Gavilán 1996). However, the alternative system of morphometric measurements called the truss network system (Strauss and Bookstein 1982, Winans 1987), constructed with the help of anatomical landmark points that enhance discrimination among groups, has been less explored for population structure analysis in freshwater fish. This type of morphometric analysis has been mostly applied to Chilean marine fish (e.g. Cortés et al. 1996, Gacitúa et al. 2008), to facilitate interpretation of the biological significance of population structures in species with wide distribution ranges.

In this study we determined the level of morphological variation in three *T. areolatus* populations collected from different river basins located in southern Chile. Given that these basins present particular hydrological conditions, we hypothesized that populations differ or segregate morphologically from each other, based on the river basin that they were sampled from. In order to address these objectives, we examined several specimens of each population based on 22 morphometric distance characters, using a truss network that covers the body shape dimensions in a relatively homogeneous way. This ensured a significant amount of information about the shape of individuals was gathered. This dataset was then subject to multivariate analyses to evaluate the degree of population separation, and to identify the body regions that experienced shape variations.

## Materials and methods

### Study areas and sampling sites

**Figure 1.**
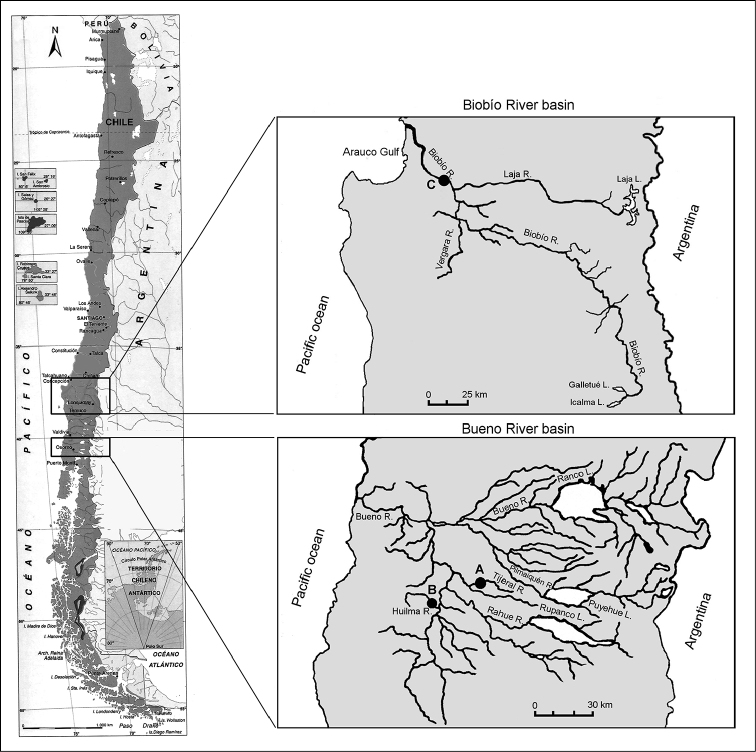
Location of sample sites of *Trichomycterus areolatus* populations located in two river basins from southern Chile. Bueno River basin: **A** Tijeral River, and **B** Huilma River; Biobío River basin: **C** Biobío River.

Specimens were collected from the Tijeral (TIJ) (n = 22) (40°37'S; 73°02'W) and Huilma (HUI) (n = 32) (40°43'S; 73°13'W) Rivers in the Bueno River basin, Province of Osorno, 10th Region; and from the Biobío River (BIO) (n = 50) (37°11'S; 72°47'W) in the Biobío River basin, Province of Biobío, 8th Region (Figure 1). These basins are located in southern Chile and originate in the western Andean Mountains at altitude above 1,000 meters and flow in a relatively straight line until reaching the Pacific Ocean. The basins are separated by about 500 km from north to south. The Biobío River basin (36°43'–38°55'S, 70°49'–73°10'W) has a drainage area of 24,029 km^2^ and represents Chile’s third largest river basin. It has a length of about 400 km and a mean flow of 900 m^3^/s (Errazuriz et al. 2000). This basin shows marked changes in flow between seasons, from 391 to 3,697 m^3^/s (Dirección General de Aguas 2004a). From a hydrological perspective, the basin is nival and rapidly-filling, and its rivers are classified as torrential with a mixed regime. Local climatic conditions are warm-temperate with winter rains. Mean annual precipitation reaches from 730 mm to 1,072 mm, and the mean annual temperature is 14.7 °C (Errazuriz et al. 2000). The Bueno River basin (39°53'– 41°23'S, 71°43'–73°15'W) has a drainage area of 17,210 km^2^ and a length of about 200 km. The mean flow is 570 m^3^/s (Errazuriz et al. 2000), ranging from 346 and 1106 m^3^/s (Dirección General de Aguas 2004b). Hydrological characteristics of the basin include a constant flow and weak slope, and its rivers are classified as quiet rivers with a lacustrine regulation. The climate is warm-temperate and rainy with Mediterranean influence. Mean annual precipitation and mean annual temperature is 2,490 mm and 12.0 °C, respectively (Errazuriz et al. 2000). In addition, the Biobío River basin has four-fold more suspended solids than the Bueno River basin (2,157 vs. 485 tons/month) (Brown and Saldivia 2000). Specimens were collected in September, October and December 2002, and March 2004 using two-pass electrofishing from 100 m of river bed, mainly in areas with small substrates and shallow water. The specimens were anesthetized with a lethal dose of benzocaine before identification as *T. areolatus* based on diagnostic characters as described by Arratia (1981). After identification, the specimens were fixed and deposited in the fish collection of the Laboratorio de Biología Molecular y Citogenética of the Universidad de Los Lagos, Osorno, Región de Los Lagos (LBMULA), under identification numbers LBMULA 363–366, LBMULA 369–375, LBMULA 382–386, LBMULA 388–412 and LBMULA 414–433. Moreover, dorsal fin rays were also counted, as an additional diagnostic character for enhanced differentiation this species from other trichomyterid catfish possibly distributed in southern Chile (e.g. *Hatcheria macraei* (Girard, 1855)), as has been suggested by Unmack et al. (2009b). Counts revealed that the specimens from all populations studied had the expected number of dorsal fin rays (TIJ= 6–8, HUI= 6–9, BIO= 4–8) for the species (Unmack et al. 2009b).

### Morphometry procedure

**Figure 2.**
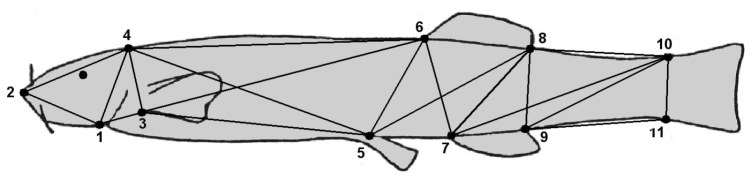
Position of the anatomical landmarks used to measure the size of 22 morphological characters on *Trichomycterus areolatus* based on a truss network. Definition of each character and its classification according to body shape dimension covered by them was as follows: **a** Head length, 1–2 = ventral tip of the operculum to tip of the head, 2–4 = tip of the head to posterior margin of the head **b** Head depth, 1–4 = ventral tip of the operculum to posterior margin of the head, 3–4 = base of the pectoral fin to posterior margin of the head **c** Anterior body length, 1–3 = ventral tip of the operculum to base of the pectoral fin, 3–5 = prepelvic length; 4–5 = posterior margin of the head to base of the pelvic fin; 3–6 = base of the pectoral fin to anterior base of the dorsal fin; 4–6 = predorsal length **d** Middle body depth, 5–6 = base of the pelvic fin to anterior base of the dorsal fin, 5–8 = base of the pelvic fin to posterior base of the dorsal fin, 6–7 = anterior base of the dorsal fin to anterior base of the anal fin, 7–8 = anterior base of the anal fin to posterior base of the dorsal fin **e** Middle body length, 5–7 = base of the pelvic fin to anterior base of the anal fin, 6–8 = dorsal fin base length, 7–9 = anal fin base length **f** Posterior body length, 7–10 = anterior base of the anal fin to dorsal posterior margin of the caudal peduncle, 9–10 = posterior base of the anal fin to dorsal posterior margin of the caudal peduncle **g** Peduncle depth, 8–9 = anterior caudal peduncle depth, 10–11 = posterior caudal peduncle depth **h** Peduncle length, 8–10 = dorsal caudal peduncle length, 9–11 = ventral caudal peduncle length.

Twenty-four morphometric characters were analyzed, including two conventional characters, total length and standard length, and 22 distance characters derived from a truss network constructed by interconnecting eleven landmarks representing the basic shape of the fish (Figure 2). The landmarks were selected for this particular fish species according to Winans (1987): 1) ventral tip of the operculum, 2) the most distal point of the head, 3) base of the pectoral fin, 4) posterior margin of the head, 5) base of the pelvic fin, 6) anterior base of the dorsal fin, 7) anterior base of the anal fin, 8) posterior base of the dorsal fin, 9) posterior base of the anal fin, 10) dorsal posterior margin of the caudal peduncle and 11) ventral posterior margin of the caudal peduncle. Several distance characters considered in this study covered the head and caudal regions of fish; these body areas are particularly interesting to analyze because, according to previous studies (Arratia 1982, 1983, Pardo 2002), *T. areolatus* populations may exhibit important morphological variation in these body regions. Truss measurements were performed manually on whole fixed specimens using a digital caliper with a precision of 0.01 mm. To reasonably eliminate any variation attributable to allometric growth, all measurements were standardized following Elliott et al. (1995), according to the following equation:

*M_adj_ = M* (*L_S_ ∙ L_O_^-1^*)*^b^*

where *M_adj_* is the size adjusted measurement, *M* is the original measurement, *L_S_* is the overall mean of the standard length (SL) for all fish from all samples in each analysis and *L_O_* = is the SL of the fish. Parameter *b* was estimated for each character from the observed data as the slope of the regression of log *M* on log *L_O_*.

### Multivariate data analysis

Prior to statistical analysis the variables were analysed for conformance to assumptions regarding normal distribution and homogeneity of variance using the Kolmogorov–Smirnov (K-S) and Levene´s tests, respectively. Differences among populations were tested with an analysis of variance (ANOVA), either with parametric (one-way ANOVA) or non-parametric (Brown–Forsythe test) tests, using each character as a response variable. The Brown–Forsythe test was applied, given that some variables presented a heterogeneous variance among groups. The transformed data were subjected to principal component analysis (PCA) and discriminant function analysis (DFA) to evaluate any phenotypic differences among populations. Individual scores from PCA were used to construct a scatterplot to reveal the specimen groupings. The eigenvectors and eigenvalues were obtained from the PCA correlation matrix, which allowed the largest part of the variance of original variables to be reduced to a small number of components. The analysis evaluates the relationships among populations according to their proximity in the space defined by the components. Thus, plotting the component scores of specimens can reveal natural groupings, without *a priori* knowledge of such groupings. The significance of the separation among groups was determined using an analysis of similarity (ANOSIM) test. This test is a generalization of the univariate ANOVA and it has the property to consider all variables during the calculation of similarity among populations based on the Euclidean distance matrix. In this test, *r*-values range from 0 to 1, where 0 indicates no separation of groups and 1 corresponds to complete discrimination between groups (Clark 1993). Only 22 truss measurements were included in the PCA analyses. The number of principal components useful for this analysis was determined by using the Parallel Analysis (PA) based on the retaining of PCA eigenvalues from the data greater than PA eigenvalues from the corresponding random data (Franklin et al. 1995). Significant different (*p* <0.01) truss measurements were further subjected to DFA for case classification using separate covariance matrix. This method is recommended to address the problem of inequality of covariance matrices among groups in DFA (Anderson and Bahadur 1962). The Wilks’s λ was used to compare the differences among all groups. The ability of the phenotypes to discriminate among populations was assessed with a cross-validation test. This required the removal of one individual from the original matrix, and then a discriminant analysis was performed with the remaining observations to classify the omitted individual. Performance was evaluated according to the percentage of correctly and incorrectly classified fish. The morphological distinctness of the population was defined as the percentage of correctly-classified individuals. Kolmogorov–Smirnov, ANOVA, Brown–Forsythe, and DFA analyses were carried out using SPSS v. 19 (IBM Corp., Armonk, NY, USA), while PCA and ANOSIM analyses were performed with MATLAB R2010a (The MathWorks, Inc.) and PAST v. 3.14 (Hammer et al. 2001), respectively.

## Results

Table 1 shows the average values ​​of TL, SL and the 22 truss measurements analyzed. Nineteen of 22 truss measurement were found to be significantly different among populations (Table 1), including 17 (1–2, 1–3, 1–4, 2–4, 3–4, 3–6, 4–6, 5–6, 5–7, 6–7, 6–8, 7–9, 7–10, 8–9, 8–10, 9–10, 9–11) with highly significant (*p* < 0.01) values that were further tested in multivariate analysis using DFA.

**Table 1.** Morphometric data for 24 characters of three *Trichomycterus areolatus* populations from southern Chile.

Character	Tijeral River (Mean ± SD) (n = 22)	Huilma River (Mean ± SD) (n = 32)	Biobío River (Mean ± SD) (n = 50)	ANOVA (Exact *p*-value)
Total length, TL (cm)	7.02 ± 1.64	6.66 ± 0.92	6.16 ± 1.24	0.021*
Standard length, SL (cm)	6.27 ± 1.53	5.66 ± 0.75	5.51 ± 1.17	0.036*
Truss measurements (cm)				
1–2	1.04 ± 0.29	0.96 ± 0.12	0.58 ± 0.10	<0.001***(§)
1–3	0.25 ± 0.12	0.17 ± 0.08	0.46 ± 0.08	<0.001***
1–4	0.63 ± 0.16	0.59 ± 0.11	0.49 ± 0.08	<0.001***(§)
2–4	0.80 ± 0.19	0.74 ± 0.14	0.84 ± 0.14	<0.001***(§)
3–4	0.73 ± 0.18	0.63 ± 0.13	0.53 ± 0.09	<0.001***(§)
3–5	1.82 ± 0.71	1.80 ± 0.30	1.94 ± 0.39	0.024 *(§)
4–5	2.51 ± 0.56	2.29 ± 0.35	2.20 ± 0.44	0.858 ^NS^
3–6	2.57 ± 0.69	2.32 ± 0.37	2.48 ± 0.53	<0.001***
4–6	2.89 ± 0.70	2.60 ± 0.42	2.65 ± 0.54	<0.001***
5–6	0.96 ± 0.28	0.77 ± 0.15	0.96 ± 0.26	<0.001***
5–7	0.99 ± 0.39	0.74 ± 0.30	0.93 ± 0.23	0.004 **(§)
6–7	0.94 ± 0.24	0.81 ± 0.16	0.77 ± 0.17	0.007 **
5–8	1.56 ± 0.48	1.48 ± 0.29	1.36 ± 0.29	0.053^NS^
6–8	0.85 ± 0.27	0.89 ± 0.21	0.61 ± 0.13	<0.001***(§)
7–8	0.75 ± 0.26	0.69 ± 0.16	0.66 ± 0.12	0.929 ^NS^(§)
7–9	0.33 ± 0.15	0.36 ± 0.12	0.41 ± 0.10	<0.001***(§)
8–9	0.53 ± 0.19	0.42 ± 0.08	0.49 ± 0.10	<0.001***(§)
7–10	1.86 ± 0.53	1.63 ± 0.24	1.84 ± 0.42	<0.001***
8–10	1.30 ± 0.39	1.0 ± 0.19	1.44 ± 0.32	<0.001***
9–10	1.40 ± 0.38	1.15 ± 0.17	1.36 ± 0.29	<0.001***
9–11	1.22 ± 0.36	0.98 ± 0.20	1.25 ± 0.28	<0.001***
10–11	0.57 ± 0.16	0.48 ± 0.09	0.48 ± 0.10	0.040 *

* *p* < 0.05

** *p* < 0.01

*** *p* < 0.001

§ Significance from Brown–Forsythe test

n = sample size

NS = not significant

**Figure 3.**
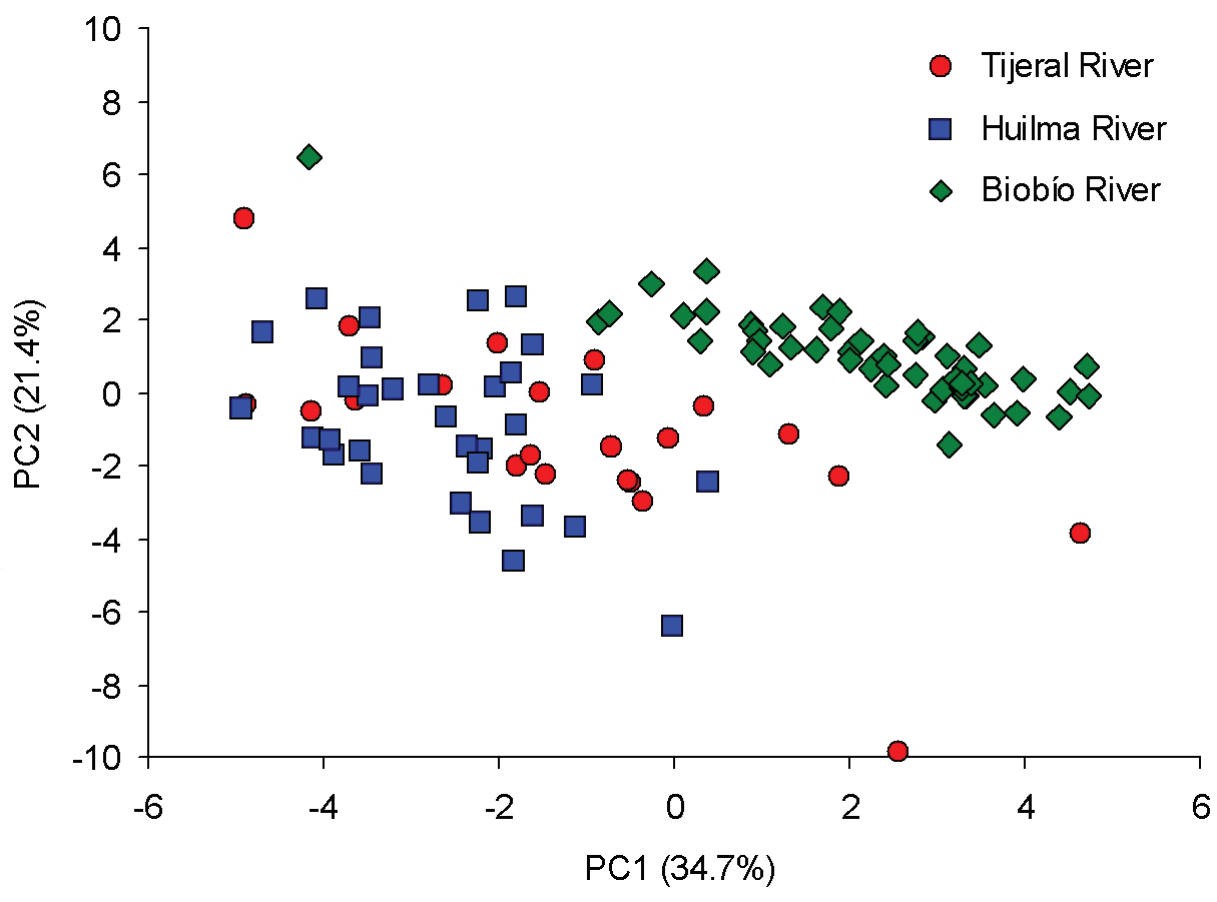
Scatterplot for individual scores from Principal Component Analysis (PC1 on PC2) of three *Trichomycterus areolatus* populations from southern Chile according to 22 truss measurements derived from a truss network.

The PCA based on 22 truss measurements retained two components according to PA, explaining 56.16% of the total variance. The first (PC1) and second (PC2) principal components accounted for 34.73 and 21.44% of the total variance, respectively (Table 2). Thus, PC1 was the most important component contributing to separation among populations. These differences were primarily because of the strong loading of 1–3, 2–4, 3–5, 3–6, 5–6, 5–7, 7–9, 8–9, 7–10, 8–10, 9–10, and 9–11 characters. Most of these characters were involved in longitudinal body shape changes (i.e., shape changes corresponding to the anterior-posterior body that reflect length changes) either at the head (2–4), anterior body (1–3, 3–5 and 3–6) or caudal peduncle (8–10 and 9–11) regions. Strong loading of characters involved in body depth shape variation (i.e., corresponding shape changes of the dorsal-ventral body axis) at the dorsal fin in the middle body (5–6) and caudal peduncle (8–9) regions, were also observed (Table 2). In the case of PC2, within the eight characters that exhibited strong loadings, most were associated to body depth shape variation either at head (1–4 and 3–4), dorsal fin in the middle body (6–7, 5–8 and 7–8) or caudal peduncle (10–11) regions. The scatter-plot of PC1 and PC2 scores for each sample revealed no overlapping (TIJ and HUI vs. BIO) or some overlapping (TIJ vs. HUI) dot clusters among *T. areolatus* populations (Figure 3). There were highly significant difference between populations based on the ANOSIM test (*r* = 0.552, *p* < 0.0001).

**Table 2.** Component loadings of the first two principal components derived from PCA based on the correlation matrix of 22 truss measurements of *Trichomycterus areolatus* populations from southern Chile. Characters of greater contribution on each component are in bold.

		Component	
		PC1	PC2
Eigenvalue		7.640	4.716
Explained variance (%)		34.728	21.438
Cumulative variance (%)		34.728	56.166
Character	Body shape dimension		
1–2	Head length	-0.177	-**0.341**
1–3	Anterior body length	**0.324**	0.046
1–4	Head depth	-0.148	-**0.282**
2–4	Head length	**0.209**	0.134
3–4	Head depth	-0.126	-**0.307**
3–5	Anterior body length	**0.250**	-0.130
4–5	Anterior body length	-0.062	0.031
3–6	Anterior body length	**0.253**	0.039
4–6	Anterior body length	0.159	-0.002
5–6	Middle body depth	**0.237**	-0.083
5–7	Middle body length	**0.222**	-0.170
6–7	Middle body depth	0.066	-**0.293**
5–8	Middle body depth	0.060	-**0.382**
6–8	Middle body length	-0.109	-**0.381**
7–8	Middle body depth	0.096	-**0.346**
7–9	Middle body length	**0.226**	-0.139
8–9	Peduncle depth	**0.239**	-0.180
7–10	Posterior body length	**0.259**	-0.036
8–10	Peduncle length	**0.324**	0.080
9–10	Posterior body length	**0.314**	0.036
9–11	Peduncle length	**0.310**	0.045
10–11	Peduncle depth	0.127	-**0.274**

**Figure 4.**
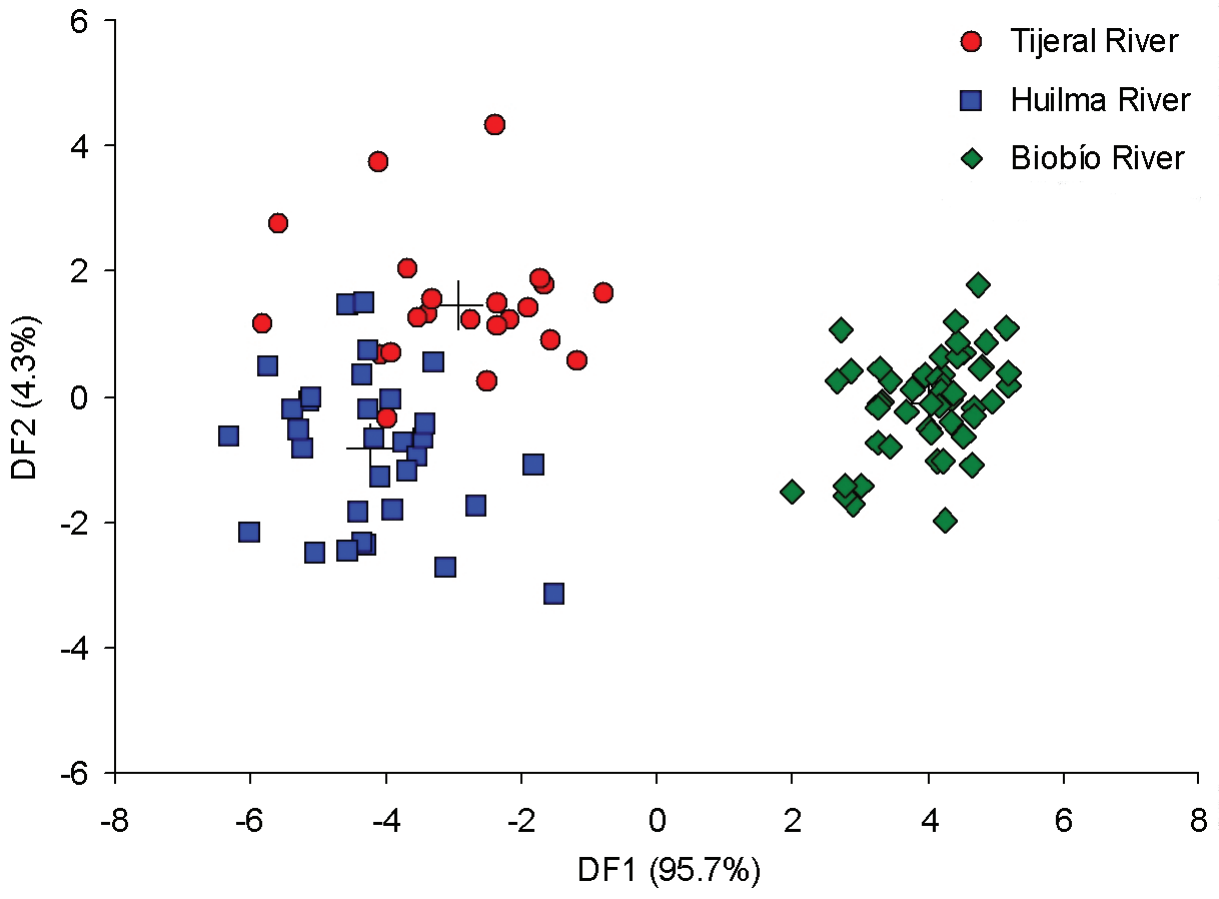
Scatterplot for individual scores from Discriminant Function Analysis (DF1 on DF2) of three *Trichomycterus areolatus* populations from southern Chile according to 17 truss measurements derived from a truss network. Crosses indicate group centroids.

The DFA based on 17 truss measurements with highly significant (*p* < 0.01) differences among populations produced two discriminant functions. The first (DF1) and second (DF2) discriminant functions explained 95.7% and 4.3% of the total variance, respectively, together accounting for 100% of the morphological variation. This result was supported by the high canonical correlations among the discriminant functions and groups, which had values of 0.969 y 0.641 for the first and second functions, respectively. Furthermore, both DF1 and DF2 generated statistically significant differences among the groups (Wilks’s λ = 0.036, χ^2^ = 309.803, d.f. = 34, *p* < 0.01). This result indicated significant morphological differences among the three populations. The DF1 vs DF2 scatter-plot revealed a clear separation among the point clouds for the three populations (Figure 4), a result that was consistent with the clusters observed in the PCA scatter-plot. The structure matrix (Table 3), which shows the intra-group correlations between each of the characters and the discriminant functions, revealed 17 truss measurements with high correlations. The variables with meaningful loading on DF1 were 1–2, 1–3, 6–8, 2–4, 3–6, 1–4, 7–10 and 4–6, while on DF2 they were 9–10, 8–9, 8–10, 3–4, 5–7, 9–11, 7–9, 6–7 and 5–6, showing that these characters were mainly responsible for differences among the populations. In DF1 all variables represent measurements covering the entire body of the fish; in contrast, these were concentrated mostly in the tail region in DF2.

Discriminant function analysis showed 93.3% correct classification of individuals into their original populations, and the cross-validation test produced comparable results (92.3%) (Table 4). The percentage of correctly-classified fish was highest in all populations, with 100% in BIO, 81.8% in TIJ and 90.6% in HUI. The last two populations included a slight mixture of individuals from each other.

**Table 3.** Structure matrix coefficients that show the intra-group correlations between each of the characters and the discriminant functions. Characters of greater contribution in each discriminant function are in bold.

Character	Body shape dimension	Function	
		DF1	DF2
1–2	Head length	-**0.428**	0.353
1–3	Anterior body length	**0.390**	0.142
6–8	Middle body length	-**0.227**	-0.173
2–4	Head length	**0.220**	-0.152
3–6	Anterior body length	**0.190**	-0.071
1–4	Head depth	-**0.149**	0.121
7–10	Posterior body length	**0.139**	0.102
4–6	Anterior body length	**0.121**	0.014
9–10	Posterior body length	0.234	**0.378**
8–9	Peduncle depth	0.116	**0.376**
8–10	Peduncle length	0.263	**0.372**
3–4	Head depth	-0.183	**0.356**
5–7	Middle body length	0.069	**0.332**
9–11	Peduncle length	0.217	**0.311**
7–9	Middle body length	0.109	-**0.290**
6–7	Middle body depth	-0.054	**0.285**
5–6	Middle body depth	0.121	**0.223**

**Table 4.** Percentage of *Trichomycterus areolatus* specimens from populations of southern Chile correctly classified into their original group and after cross-validation.

Group	Population	Tijeral River	Huilma River	Biobío River	Total
Original (%)†	Tijeral River	81.8	18.2	0.0	100
	Huilma River	9.4	90.6	0.0	100
	Biobío River	0.0	0.0	100	100
Cross-validated (%)‡	Tijeral River	77.3	18.2	4.5	100
	Huilma River	6.3	90.6	3.1	100
	Biobío River	0.0	0.0	100	100

† The 93.3% of originally grouped cases were correctly classified

‡ The 92.3% of cross-validated grouped cases were correctly classified

## Discussion

The results of this study obtained from the truss-based morphometrics indicated that *T. areolatus* from southern Chile showed significant phenotypic heterogeneity among the populations. Both multivariate analyses using PCA and DFA suggested three distinct phenotypic populations of *T. areolatus*. The segregation among populations was confirmed by PCA and DFA scatter-plot based on scores for each sample that showed non-, or slight, overlapping clusters of points for each population. This result was also supported by the percentage of correctly-classified individuals, where 93.3% of individuals showed correctly classification into their respective groups by DFA, indicating low intermingling among the populations. Populations of the Tijeral plus Huilma Rivers versus the Biobío River showed non-overlapping, possibly due to the large distances among drainages. This was not the case in the morphological parameters of populations of the Tijeral River versus the Huilma River that showed some overlapping between populations, which may be attributed to the small geographic distances between them given that both belong to the same drainage in southern Chile (Bueno River basin) and probably share more similar environmental conditions. Our results are similar to those of Pardo (2002) who reported morphometric variation based on the geometric morphometric technique in two *T. areolatus* populations from different river basins of south-northern Chile. Our results also agree with Arratia (1982, 1983) who reported phenotypic heterogeneity in this species, particularly, among populations collected across central Chile, although the differences were found in the bones of the caudal skeleton.

The PCA confirmed that the variation in morphological measurements of the study populations of *T. areolatus* involved several characters related to the head region, body depth, and caudal peduncle. For example PC1, which was the most important component contributing to separation between populations (34.72% of the total explained variance), presented twelve characters with strong loading on this component, associated with body length and depth. For their part, PC2 that was also an important component (21.44% of the total explained variance), showed characters mainly associated to body depth variation in the head region, including the caudal peduncle region. Thus, the morphological variation registered in *T. areolatus* populations by PCA revealed changes on several body regions and/or dimensions.

Understanding the origin of morphological differences between populations of *T. areolatus* is challenging. This is because fish morphology is a complex phenotype that is determined by genetics and environment factors, and the interaction between them (Poulet et al. 2004, Keeley et al. 2006, Schwander and Leimar 2011, Colihueque and Araneda 2014). In addition, other forces such as ontogeny, performance, fitness and behavior may also change the body shape (Walker 2010). Thus, for example, phenotypic variability among populations may arise without major genetic differentiation as a consequence of the isolation of portions of a population within local habitats or when they occupy heterogeneous habitats across their distribution range. In other cases, genetic differentiation among populations precedes phenotype divergence (Schwander and Leimar 2011). It is also possible that preexisting genetic differences among current populations can be enhanced by their isolation, resulting in a notable inter-population structuring (Esguícero and Arcifa 2010). Previous molecular studies of *T. areolatus* across its distribution range reveal a high level of genetic differentiation among populations within and between watersheds (Unmack et al. 2009a, Quezada–Romegialli et al. 2010). This high degree of genetic divergence seem to be related to geographic isolation and the subsequent low genetic flow among populations of this species, given that Chilean watersheds flow relatively straight from the east (Andean mountains) to the west (Pacific Ocean), limiting opportunities for contact despite relatively short geographical distances among populations. Thus, the morphological variations observed in the present study for *T. areolatus* might be due to genetic differences among the populations. In natural populations of other fish species, the association between genetic differentiation and morphology variation among populations has been well-supported. In particular, Keeley et al. (2006) demonstrated that differences in external morphological traits among rainbow trout, *Oncorhynchus mykiss* (Walbaum, 1792) populations had a significant genetic component. In addition, these authors proposed that this morphological distinction could be, at least in part, a response to natural selection in contrasting environments. Taylor et al. (2011) after analyzing several populations of the same species from British Columbia, came to a similar conclusion, since they observed a significant positive correlation between genetic and morphological divergence among populations.

The morphometric variations observed among different populations of *T. areolatus* in the present study might also be associated with phenotypic plasticity in response to the different environmental factors of various habitats. Included within these factors are the hydrological characteristics of rivers, given that available data reveal an important influence of some hydrological parameters on body shape variation in fishes. For example, several studies have shown that water velocity can modify body shape in various fish species, such as salmonids (Pakkasmaa and Piironen 2000), Caspian cyprinid, *Alburnus chalcoides* (Güldenstädt, 1772) (Bagherian and Rahmani 2009) and the Indian major carp, *Labeo rohita* (Hamilton, 1822) (Mir et al. 2013). Differences in the water current have also been cited as an important factor that can affect body shape variation in Brazilian epigean and hypogean *Ancistrus* catfishes (Reis et al. 2006). In addition, experimental data on the brook charr, *Salvelinus fontinalis* (Mitchill, 1814) (Imre et al. 2002), the Arctic charr, *Salvelinus alpinus* (Linnaeus, 1758) (Grünbaum et al. 2007), the rainbow trout (Keeley et al. 2006), and gibbose centrarchid species (Istead et al. 2015) also revealed that changes in water velocity may affect the morphology of different areas of fish body, such as head depth and length, caudal peduncle depth, caudal fin depth and length, body depth, pelvic fin and dorsal fin lengths. These findings provide strong support that the hydrological conditions of the rivers may play an important role in morphology variations in fishes. Of note is that the study populations of *T. areolatus* belong to different basins in southern Chile (Biobío River basin and Bueno River basin) whose hydrological conditions are dissimilar with respect to various parameters, such as, quantity of water flow, turbidity and temperature (Dirección General de Aguas 2004a,b). In addition, Biobío River basin experiences significantly more variable water flow throughout the year than Bueno River basin, resulting in rapid filling during various periods of the year, and therefore, exhibiting more turbulent water conditions than Bueno River basin (Errazuriz et al. 2000). Future controlled or field experiments should be undertaken to reveal the degree of body shape variation of *T. areolatus* populations from southern Chile that could be attributed to environmental variability among rivers. This variability is may be related to water current velocity given that habitats with high water current are preferentially used by this species, compared with other sympatric species (Garcia et al. 2012).

The truss-based morphometrics represent a system of morphometric measurements that enhance discrimination between group, based on a systematic detection of body shape differences in both diagonal and horizontal and vertical directions (Strauss and Bookstein 1982, Winans 1987). One of the properties of this system is to ensure uniform coverage of the landmark configuration, being able to capture information about the shape of an organism. This class of morphometric analysis has been less explored in catfish, in spite of its potential to facilitate interpretation of morphology variation through multivariate analisis, such as PCA. In our case, the truss-based system showed a high performance to distinguish *T. areolatus* populations based on morphological data, and also to determine the specific body shape characters that contributed to such variations.

In conclusion, the findings of this study based on truss-based morphometrics indicated significant morphological variations among three *T. areolatus* populations from southern Chile. Thus, these results suggest an underlying population structure of the species.
